# The exposome impact on hair health: etiology, pathogenesis and clinical features ‒ Part I^☆^

**DOI:** 10.1016/j.abd.2024.07.003

**Published:** 2024-11-17

**Authors:** Stephano Cedirian, Ludmila Prudkin, Bianca Maria Piraccini, Julia Santamaria, Jaime Piquero-Casals, David Saceda-Corralo

**Affiliations:** a Dermatology Unit, IRCCS Azienda Ospedaliero-Universitaria di Bologna, Policlinico S. Orsola-Malpighi, Bologna, Italy; b Department of Medical and Surgical Sciences, Alma Mater Studiorum University of Bologna, Italy; c Innovation and Development, ISDIN, Barcelona, Spain; d Department of Dermatology, Clínica Dermatológica Multidisciplinar Dermik, Barcelona, Spain; e Servicio de Dermatología, Hospital Universitario Ramón y Cajal, Madrid, Spain; f Trichology Unit, Grupo de Dermatología Pedro Jaén, Madrid, Spain

**Keywords:** Exposome, Humidity, Plastic surgery procedures, Ultraviolet rays

## Abstract

Human hair, particularly on the scalp, serves as a significant aspect of social identity and well-being. The exposome, encompassing both intrinsic and extrinsic factors, plays a fundamental role in hair weathering. Intrinsic factors include genetic predispositions and physiological changes within the body, while extrinsic factors comprise environmental exposures such as UV radiation, pollution, humidity, temperature variations, lifestyle choices, and chemical treatments. These elements collectively contribute to the cumulative damage experienced by hair over time. Understanding the comprehensive impact of the exposome on hair health and hair aging necessitates an exploration of various environmental conditions, lifestyle factors, and technical artifacts. Despite advancements in research, the intricate mechanisms underlying the exposome influence on hair remain incompletely understood. Through a comprehensive review of current literature and emerging research findings, this study aims to enhance the understanding of exposome impact on hair health.

## Introduction

Human hair, particularly on the scalp, holds immense significance in social interactions and personal identity, deeply impacting one's sense of well-being.^1^ At the core of hair structure are the hair shaft and hair follicle (HF). The hair shaft comprises three primary components: the cuticle, cortex, and medulla. The cuticle acts as a protective shield for the hair, guarding it against chemical and mechanical harm. The cuticle consists of overlapping scales made of keratinocytes. Interestingly, the thickness and number of these scales vary across different ethnicities. For instance, individuals of Asian descent typically have the thickest cuticles, followed by Caucasians, with those of African descent having the thinnest cuticles. This variation affects the susceptibility of hair to breakage, with African hair being more prone due to its thinner cuticle layer. Additionally, the common practice of straightening hair in this population significantly contributes to hair breakage.^2^, ^3^, ^4^ Within each cuticle cell lies a thin protein membrane known as the epicuticle, which is crucial for maintaining the hydrophobicity of the hair. Beneath the cuticle lie three distinct layers: the A-layer, the exocuticle or B-layer, and the endocuticle. These layers are primarily composed of proteins rich in cysteine, which provide strength and structure to the hair shaft through cross-links. The A-layer contains the highest concentration of cystine, contributing significantly to the hair's resilience. The beta-layer, a primary component of the cell membrane complex (CMC), is particularly susceptible to damage from chemical treatments such as bleaching, dyeing, and straightening procedures.^2^, ^3^, ^4^ The cortex, constituting the bulk of the hair shaft, is responsible for the hair's mechanical strength and resilience. It contains elongated cells arranged in a fibrous matrix and melanin granules, which determine hair color. Within the skin, the HF serves as the primary growth structure for human hair, comprising outer and inner root sheaths, a hair bulb, and an infundibulum.^5^ HFs undergo continual cycles of change throughout life, transitioning through active growth (anagen), regression (catagen), and a resting phase (telogen). With approximately 100,000 mature HFs on the scalp, even minor alterations in their cycling patterns can hold significant clinical implications.^6^

Hair weathering is significantly influenced by exposomal factors. The exposome encompasses all exposures individuals encounter from birth to death, including external and internal elements, as well as the body's responses to them.^7^ Continuous exposure to such elements can induce hair weathering, leading to hair thinning, breaking, weathering, and overall premature aging of the hair.^8^, ^9^, ^10^ Key exposomal elements impacting hair weathering include environmental conditions (humidity, temperature, wind speed, and Particulate Matter (PM) density), spatial variables, lifestyle factors (altitude, population density), and technical artifacts (batch effects), among others.^8^, ^9^, ^11^

Nonetheless, the comprehension of how these factors influence hair weathering remains limited. This paper aims to address this limitation by thoroughly investigating the interconnection between the exposome and hair health, emphasizing it as the central focus of this study.

## Hair aging

Hair aging is a complex phenomenon, strictly related to hair weathering, influenced by various factors. Factors impacting HFs comprise racial and gender characteristics, metabolic rates, nutritional status, hormonal responses, and fluctuations in the hair growth cycle.^7^, ^8^, ^12^, ^13^ These changes encompass not only alterations in hair growth patterns and color but also variations in sebum secretion, hair thickness, and overall hair density, resulting in the hair appearing lackluster, gray, sparse, and frayed.^12^, ^13^, ^14^

Hair graying is commonly recognized as one of the initial signs of aging. Epidemiological research indicates that between 6% to 23% of individuals have hair that is 50% gray by the time they reach 50 years of age. However, the onset of graying is influenced by genetic factors, ethnicity and geographic origin. For instance, Asians and Africans typically experience later onset and less pronounced graying compared to Caucasians, with respective ages of onset averaging around 43.9 ± 10.3 years and 34 ± 9.6 years.^13^, ^14^, ^15^ The pigmentation of HFs is governed by intricate interactions among follicular melanocytes, matrix keratinocytes, and fibroblasts. Unlike the continuous melanin production observed in epidermal pigmentation, follicular melanogenesis relies on follicular cycling, with melanin not being produced during telogen, which is extended in older individuals. Hair graying occurs due to a reduction in both the population of follicular melanocytes and melanin content, exacerbated by diminished capacity to withstand oxidative damage.^12^, ^14^, ^15^

While hair loss and graying are the most commonly noted visible manifestations of hair aging, other changes in hair characteristics have also been documented. Reduction in hair diameter is another significant aspect of the aging process, typically starting from the age of 40 for women and from the age of 18 for men. Decreased sebum production and loss of hair luster, partly attributed to increased fiber curvature, have also been observed. In women, this decline usually begins after menopause, whereas in men, it becomes noticeable much later, around the age of 80.^12^, ^14^

In a recent investigation undertaken by Panhard et al., findings revealed that the onset and pace of hair graying are influenced by both ethnicity and geographic origin. Individuals of African-American, Thai, and Chinese heritage with darker hair shades demonstrate a diminished frequency and intensity of graying in contrast to individuals of similar ages with lighter hair tones, such as Caucasians.^16^, ^17^, ^18^, ^19^

## Exposomal factors, hair weathering and hair aging

**Fig. 1:**
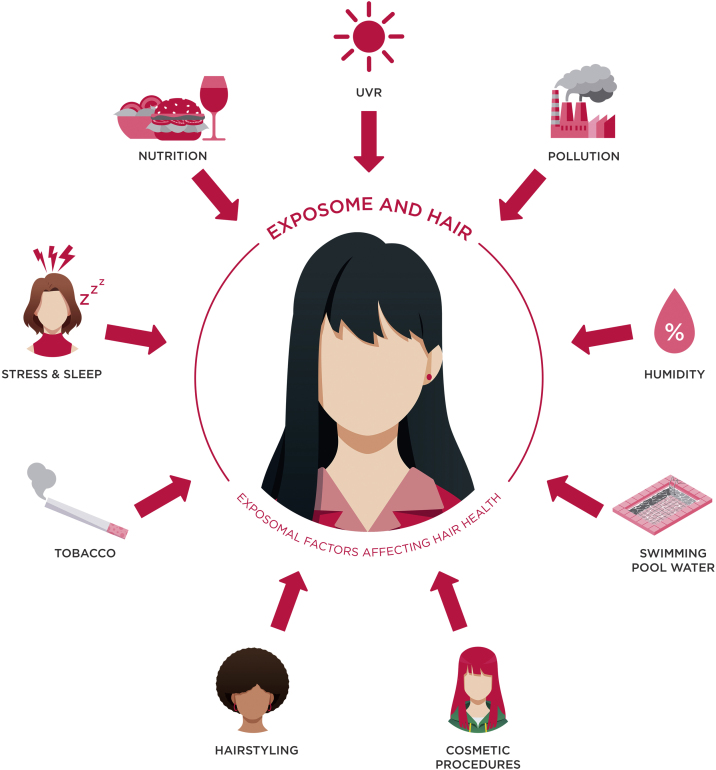
A summary image of the exposomal factors that can impact hair health.

To gain a deeper insight into the influences of the exposome on hair weathering and how it affects hair aging, it is essential to highlight that both internal and environmental exposomal factors contribute to it (Fig. 1). In the following discussion, a detailed analysis of both these classifications is provided.

### Internal exposomal factors

#### Nutrition

Nutrition plays a crucial role in maintaining optimal hair growth, and imbalances in nutrient intake can hasten the aging process of hair. Keratin, the primary component of hair, relies heavily on adequate protein intake for the production of healthy hair strands. Conversely, carbohydrates serve as a significant energy source for the body, including the HFs. The rate of cell division in HFs is influenced by the calorific value of the diet, which is largely derived from carbohydrates. These carbohydrates are stored as glycogen in the outer root sheath of the HF, providing the necessary energy for hair growth.^20^, ^21^, ^22^ Conditions such as marasmus and kwashiorkor, which are associated with protein-energy malnutrition, can lead to hair becoming fine or brittle, accompanied by alopecia triggered by telogen effluvium (TE). Kwashiorkor, characterized by inadequate protein intake despite sufficient calorie consumption, presents additional hair-related changes, including hypochromotrichia and the distinctive “flag sign” in scalp hair, indicating significant nutritional deficiencies affecting hair color and texture.^20^, ^21^, ^22^

In terms of vitamins and trace minerals, an adequate intake is essential for the biosynthesis and energetic metabolism of HFs. Specific vitamins (A, E, B group, and D) and minerals (iron, zinc, selenium, etc.) play pivotal roles in maintaining hair health, and low intakes can be associated with hair aging.^22^, ^23^ Chang et al.'s systematic review analyzed serum levels of iron, zinc, copper, and calcium in premature hair graying (PHG), revealing lower copper and calcium levels among PHG patients, critical for melanogenesis.^24^ Zinc is highlighted as a crucial micronutrient for hair growth and maintenance, as its deficiency, whether genetic or acquired, can cause alopecia^22^; However, the causality remains unclear due to a lack of evidence showing improvement in hair loss or alopecia after zinc supplementation. El-Husseiny et al.'s study on an Egyptian population found slightly lower serum vitamin D levels in PHG patients compared to controls, although not statistically significant. However, low serum ferritin levels were more prevalent in PHG patients, suggesting a link between iron deficiency and PHG.^23^ Iron deficiency, a common nutritional deficiency globally, can lead to hair loss due to its vital role in hemoglobin formation, which carries oxygen to the body's tissues, including HFs. Iron deficiency reduces oxygen delivery to HFs, resulting in hair thinning and loss.^22^, ^25^

Obesity, another nutritional-linked condition, significantly influences hair aging. high-fat diets (HFD) and obesity appear to accelerate hair loss by directly impacting HF stem cells. This impact is linked to the deficiency of COL17A1, an essential component responsible for anchoring HF stem cells to the basement membrane, leading to HF miniaturization and thinning, exacerbating the aging process of HFs. Experimental evidence from studies on mice indicates that those fed a high-fat diet exhibit noticeable hair thinning and reduced hair density compared to those on a normal diet.^26^, ^27^ The influence of obesity on hair aging extends beyond HF stem cell depletion. Metabolic stress induced by HFD alters the HF microenvironment, affecting the duration and phases of the hair growth cycle. Obesity-related metabolic stress induces premature entry into the anagen phase and disrupts the balance of the hair cycle, leading to accelerated hair thinning and loss.^26^, ^28^ Various pathways, including inflammatory cytokine signaling and oxidative stress, can contribute to hair aging due to obesity; for example, inflammatory signals like Interleukin-1β (IL-1β) inhibit Shh signaling in HF stem cells, further exacerbating hair loss.^26^

#### Stress and sleep

The human HF emerges as an unexpected site for various stress-related mediators, including corticotropin-releasing hormone (CRH), adrenocorticotropic hormone (ACTH), cortisol, noradrenaline, substance P, and prolactin. The interplay between stress mediators and HF receptors, located on diverse cell types within the HF, modulates hair cycle control and immune responses.^29^, ^30^ Specifically, the bulge region, housing epithelial and melanocyte stem cells, exhibits high receptivity to nerve-derived signals and neuroendocrine stimulations from neighboring tissues. This implies that the stem cells in the bulge have a pivotal role in assimilating signals from both blood and nerve sources to regulate hair growth and pigmentation.^29^, ^30^

Studies have illustrated that prolonged stress has the potential to hasten aging in HFs and provoke hair loss by adversely impacting the function and reproduction of HF stem cells. Corticosterone, a primary stress hormone akin to cortisol in rodents, impedes the initiation of the anagen phase in HF stem cells primarily through the corticosterone/Gas6/AXL pathway. Indeed, cortisol binds to glucocorticoid receptors (GR), resulting in the suppression of Gas6. Consequently, AXL receptors fail to receive adequate stimulation, hampering HF stem cell activation and contributing to accelerated HF aging.^14^, ^31^

Furthermore, research indicates that mitochondria play a central role in shaping the cellular response to stress, with implications for hair growth and pigmentation dynamics. Alterations in mitochondrial function and metabolism can profoundly impact the progression of the hair growth cycle, potentially connecting psychosocial stress to hair loss and PHG. Mitochondrial dysfunction induced by stress and depletion of Mitochondrial DNA (mtDNA) may contribute to irregular hair cycling and pigmentation.^29^, ^30^

Premature hair aging, hair weathering, and associated conditions are also correlated with another form of stress: sleep deprivation. Xerfan et al. elaborated on how sleep is intertwined with trichodynia through various pathways. Trichodynia, characterized by scalp discomfort and hypersensitivity, can be exacerbated by stress, triggering the release of substance P, a neurotransmitter associated with pain perception and inflammation.^32^ Substance P not only influences pain perception but also regulates circadian rhythm and sleep patterns. Disruptions in substance P levels or its interaction with neurokinin-1 receptors could result in substandard sleep quality, intensifying trichodynia symptoms.^32^ Moreover, there exists a bidirectional relationship between trichodynia and sleep disturbances: trichodynia can disturb sleep patterns, and inadequate sleep quality can heighten sensitivity to pain and discomfort, amplifying trichodynia symptoms. Additionally, disrupted sleep patterns can elevate oxidative stress levels, potentially worsening scalp sensitivity, and trichodynia symptoms, and ultimately inducing hair aging.^32^

#### Tobacco consumption

Smoking has been associated with hair weathering, alopecia and PHG.^22^, ^33^, ^34^ In AGA, the most prevalent form of hair loss, smoking is linked to hair miniaturization and the conversion of terminal hairs into vellus hairs. This phenomenon is partially attributed to the systemic influence of androgens, which can be affected by smoking. Specifically, smoking may induce a relative hypo-estrogenic state in women by hydroxylating estradiol and inhibiting aromatase, potentially impacting hair patterns influenced by androgens.^33^, ^34^ Moreover, nicotine, a constituent of tobacco smoke, can build up in hair and contribute to follicular damage; indeed, it can lead to constriction of dermal hair papilla and oxidative stress. Oxidative stress, in turn, can prompt the release of pro-inflammatory cytokines from follicular keratinocytes, which have been demonstrated to hinder HF growth in culture; free radicals can also cause DNA damage and disrupt protease/antiprotease systems. Ultimately, these mechanisms result in follicular inflammation, fibrosis, and the destruction of HFs.^33^, ^34^ Furthermore, oxidative stress can harm melanocytes, leading to reduced melanin production and, consequently, PHG. Research indicates that smokers are prone to earlier onset and a higher incidence of hair graying compared to non-smokers, with the risk of hair graying increasing with smoking duration.^33^, ^34^

### Environmental exposomal factors

#### UVR

Exposure to UV radiation triggers a cascade of chemical reactions in hair fibers that can lead to premature hair aging and hair weathering.^7^, ^8^, ^22^ Indeed, both UVA and UVB rays lead to oxidative DNA damage in HFs, reduced keratinocyte proliferation, increased apoptosis, elevated TGF‐β2 production, decreased IGF‐1 expression in outer root sheath keratinocytes, premature catagen development, and perifollicular mast cell degranulation. The severity of UV-induced HF damage is more pronounced with higher UV doses, affecting not only HF compartments near the skin surface but also extending to lower regions, including the hair bulb.^35^, ^36^ UVA radiation, in particular, contributes to the formation of stable carbon and sulfur radicals within the keratin protein structure of hair; these radicals result from the direct interaction of UV radiation with amino acid residues present in the protein matrix. Specifically, UV radiation targets amino acids like tyrosine, tryptophan, and cysteine, which are particularly susceptible to oxidation due to their chemical structure.^37^ These species are also associated with the production of oximelanin from melanin as well as the oxidation of artificial pigments in dyed hair. Melanin, the pigment responsible for hair color, plays a dual role in protecting against and succumbing to UV damage: while it absorbs and dissipates UV radiation, shielding the underlying proteins, prolonged exposure can lead to its degradation and the formation of oxidative species from it.^35^, ^36^ ROS participate in oxidative reactions that further degrade proteins and lipids, accelerating hair aging and deterioration. Additionally, UV radiation penetrates the hair shaft, causing structural changes within the keratin matrix: amino acids, sterols, and fatty acids undergo photooxidation, leading to the formation of micro-molecular lesions and weakening of the hair fiber.^35^ Prolonged exposure to UV radiation has been shown to induce notable changes in the lipid composition of hair, potentially impacting its overall health and integrity. Among the observed alterations, a decrease in vitamin A fatty acid esters and sterol esters has been documented.^38^ These lipids, crucial for HF development and maintenance, hint at possible disruptions in retinoid metabolism and lipid synthesis pathways in response to UV exposure. Ceramides, vital contributors to skin and hair barrier functions, exhibit a significant reduction following UV exposure which may contribute to compromised barrier integrity, potentially linking UV exposure to increased hair brittleness. Furthermore, the concentrations of mono-, di-, and triglycerides, commonly found in human tissues, were diminished in UV-exposed hair. This decline is attributed to oxidative processes induced by UV radiation, indicating potential lipid degradation pathways. Overall, these structural alterations manifest as increased porosity, loss of flexibility, and deterioration in hair texture.^38^

Lastly, UV radiation exposure can cause significant changes in hair color. Eumelanin and pheomelanin, the two types of melanin present in hair, undergo oxidative reactions under UV exposure, resulting in color fading, bleaching, or shifts in hue. Lighter hair colors, which contain higher levels of pheomelanin, are particularly susceptible to UV-induced color changes.^35^

#### Pollution

Pollution can also impact hair fibers, causing hair weathering. When exposed to UV radiation, hair fibers contaminated with polycyclic aromatic hydrocarbons (PAHs) become more susceptible to damage. The process involves PAHs absorbing UV light, which triggers the production of ROS within the hair fibers. These ROS, in turn, contribute to the degradation of DNA, proteins, and cell membrane components.^7^, ^8^, ^39^ This impact results in the formation of vacuoles or lacunae in the cortex and cuticle, the degradation of keratinocytes in the cortex, a decrease in cuticle layers over time, and alterations in melanosomes. Importantly, PAHs found in hair melanin intensify the adverse effects of UV radiation, potentially causing hair bleaching and subsequent PHG. Although the observed damage may not significantly compromise the overall integrity of hair fibers dominated by macrofibers, the reduction of the cuticle layer can visibly affect the hair's appearance.^39^

#### Humidity

Humidity levels play a crucial role in the formation and dynamics of radicals in hair fibers, and it can ultimately lead to hair weathering.^7^, ^8^, ^40^ High humidity conditions lead to an increase in semiquinone radical species in melanin and influence molecular assembly and radical lifetimes. This phenomenon impacts the local viscosity and molecular dynamics, thereby affecting the chemistry of reactive intermediates within the hair structure.^40^ According to experimental data, the effects of sunlight on hair proteins under high humidity generate an enhanced radical decay, involving the formation of ROS that contribute to increased protein degradation and disulfide cleavage. Indeed, when hair is exposed to UV radiation in the presence of water, a more complex series of reactions occur water acts as a facilitator, enhancing molecular mobility within the hair fiber and providing a medium for the formation of ROS, such as hydroxyl radicals.^37^ Overall, hair exposed to UV at high humidity levels exhibits increased protein loss compared to lower humidity conditions. Moreover, higher humidity levels lead to increased formation of sulfur-based radicals, which is anticipated to result in more disulfide bond cleavage in hair proteins. This phenomenon has been observed in both white and light brown hair types under UV exposure at different humidity levels.^40^

#### Swimming pool water

Chlorine water represents a significant external exposomal factor that can induce hair weathering. Specifically, chlorine, often present in the form of hypochlorous acid used to sanitize pool water, can deeply penetrate the hair cortex. Once inside, chlorine initiates oxidative processes that degrade melanin, resulting in hair discoloration over time. Moreover, chlorine exposure can expedite the breakdown of keratin within the hair structure while simultaneously compromising the integrity of the cuticle leading to hair breakage as well.^7^, ^41^ Also, repeated exposure to water, exacerbates the mechanical stress and friction experienced by hair strands; this continual friction contributes to the erosion of the cuticle, further compromising the hair's overall health and appearance.^41^ Schwartz et al. recently criticized this theory in their paper, as they propose that copper compounds in the water, binding to the protein on the surface of the hair shaft, are the real cause of green hair. Indeed, copper sulfate is frequently added to swimming pools as an algaecide. Additionally, the green tinting of previously blond hair may occur when copper pipes are new and uncoated by calcium scale.^42^ Further research is required to comprehensively understand the role of each component in the green tinting of hair undergoing continuous exposure to swimming pool water.

#### Cosmetic procedures

Cosmetic procedures undoubtedly play a significant role in daily hair care routines. However, while many of these procedures serve as bold statements of individuality, concerns have arisen regarding their potential to cause hair weathering.^7^, ^8^, ^43^, ^44^ Hair can suffer damage from both physical and chemical influences. Physical factors encompass friction, excessive combing, brushing, or exposure to heat. Chemical treatments encompass bleaching, coloring, perming, and chemical straightening. Extended and repeated exposure to these elements can alter hair texture, resulting in damage and ultimately accelerating the aging process of hair.^12^

For what concerns hair dyes, are available in two main types: oxidative (permanent) and non-oxidative (semi-permanent and temporary). Permanent dyes penetrate the hair shaft to introduce a new color through oxidation, while semi-permanent and temporary dyes adhere to the hair's outer layer and can be washed off easily. Hair dyeing can cause physical and chemical damage to both the hair shaft and the scalp.^45^, ^46^, ^47^, ^48^ The cuticle can suffer from various forms of damage such as saw-margins, fissuring, and detachment of cuticular cells. The cortex may develop irregular cell contour, cell degeneration and vacuoles in the endocuticle due to chemical reactions during dyeing. The extent of weathering depends on factors like the type of dye, application duration, chemical concentration, pH level, hair resistance, and other hair care practices.^45^, ^46^, ^47^, ^48^ Moreover, there are also chemical hazards associated with hair dyes such as allergic contact dermatitis (ACD). Dyes contain precursors, couplers, and developers that can lead to ACD. Ingredients like hydrogen peroxide (H_2_O_2_) and monoethanolamine (MEA) cause oxidative stress and cytotoxicity, contributing to hair loss and ACD.^45^, ^46^, ^47^, ^48^

Bleaching is another main procedure involved in hair cosmecosmetics associated with potential hair weathering. This technique serves as a chemical procedure aimed at lightening the inherent color of hair by oxidizing melanin. Through oxidation, melanin is depleted, resulting in lighter hair tones. This process unfolds in several stages: bleaching agents, like lightener and powder-bleach, permeate the hair cuticle and cortex to access melanin granules. Hydrogen peroxide within these agents oxidizes and disintegrates melanin granules, dismantling the pigments. During melanin granule disintegration, metallic elements within the granules are liberated and act as catalysts, hastening the bleaching process.^47^, ^49^, ^50^, ^51^ Excessive or improper bleaching can yield various adverse effects on the hair; indeed, the oxidative process in bleaching can induce deterioration of cuticle scales along with visible breaks and holes in the cuticle layer. Additionally, bleaching triggers protein loss within the hair shaft, as its oxidative nature can disintegrate keratins, leading to hair fragility and breakage.^47^, ^49^, ^50^, ^51^

Finally, hair straightening treatments and permanent weaving are also popular procedures within the hair cosmetic industry but can impact hair health. Hair straightening treatments aim to alter the mechanical properties of hair for a smoother and straighter appearance. Chemical straighteners, like those containing formaldehyde or glyoxylic acid, relax hair fibers, making them more flexible and changing their natural resistance to breakage and stretching. Conversely, physical treatments, such as using flat irons, utilize heat to modify hydrogen bonds and reconfigure the hair shaft. Both types of treatments can result in hair and scalp damage.^52^, ^53^ Chemical straighteners may cause various abnormalities in the hair shaft, including increased inter-scale distance, cuticle detachment, fissures, irregular cuticle edges, and undulations. The cuticle scale lifting can lead to greater porosity and susceptibility to weathering. Protein loss is also a concern, with formaldehyde or glyoxylic acid-based straighteners breaking down essential bonds affecting hair strength and structure. Additionally, chemical straighteners may cause scalp inflammation, irritant contact dermatitis, and ACD.^52^, ^53^

Permanent waving (PW) converts straight hair into curls through a chemical process involving breaking and restructuring disulfide bonds. The process includes breaking disulfide bonds using reducing agents, shaping the hair, a stress relaxation process (SRP), and neutralization with an oxidizing agent like hydrogen peroxide. Mercaptans like thioglycolate and glyceryl monothioglycolate are common reducing agents used in PW. Consequences of PW include reduced tensile strength, changes in proteins and lipids, increased hair friction, and difficulty in combing, as well as the potential for hair breakage and hair loss if not used correctly.^54^

#### Hairstyling

**Fig. 2:**
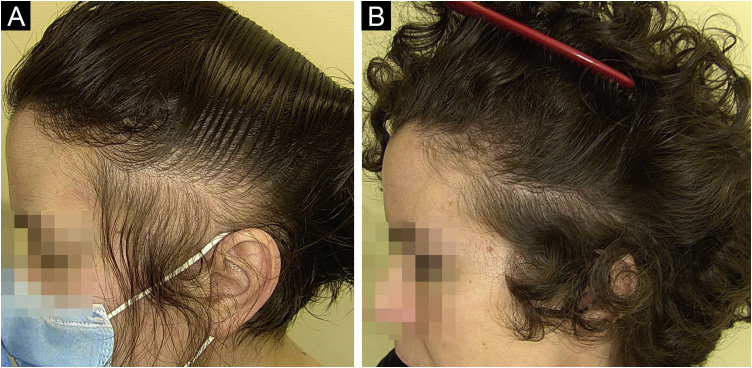
A case of traction alopecia due to a tight hairstyle, pre (A) and post (B) hair transplant.

The diverse array of modern hairstyles has enriched the landscape of hair care, yet it also brings forth potential threats to hair health. Afro hairstyles like braids, cornrows, and weaves hold deep cultural significance in African and African American communities, serving as potent symbols of identity and pride. However, these styles often involve tight braiding or weaving, leading to various hair conditions such as acquired proximal trichorrhexis nodosa (APTN), TA, traction folliculitis (TF), and central centrifugal cicatricial alopecia (CCCA).^55^, ^56^, ^57^, ^58^, ^59^ APTN is characterized by fragile hair prone to breakage from minor trauma, such as combing, tight hairstyles, or pressure during sleep. Chemical treatments or excessive brushing can exacerbate it. Trichoscopy can reveal hair breakage with Trichorrhexis nodosa, potentially causing alopecia. TA, on the other hand, results from prolonged traction on the hair due to specific hairstyles and hairpieces (e.g., weaves/extensions, tight braids/cornrows and curlers), primarily affecting black individuals. Symptoms include pain, erythema, and folliculitis. Diagnosis relies on clinical and trichoscopic findings like the flambeau sign, hair casts, and reduced hair density (Fig. 2A‒B).^55^, ^56^, ^57^, ^58^, ^59^ Lastly, CCCA is associated with sewn-in weaves and braids with extensions, primarily affecting African-descended women. It causes progressive hair loss, particularly in the central scalp region, leading to follicle destruction and scalp discomfort. Trichoscopy may display reduced hair density, hair shaft variability, small pinpoint white dots, single hairs or groups of two hairs surrounded by a peripilar white-gray halo, and pigmented asterisk-like macules with sparse terminal and vellus-like hairs.^55^, ^56^, ^57^, ^58^, ^59^

Backcombing, also known as hair teasing, is another trendy hairstyling approach where the hair is combed towards the scalp to enhance volume and texture. It's commonly employed to elevate the hair or achieve a fuller appearance, particularly for styles like up-dos or beehives.^60^ This hairstyle can cause damage if not performed correctly or too frequently; indeed, it can lead to hair breakage as strands may become tangled and knotted, resulting in breakage when attempting to detangle the teased hair. In addition, this technique can lift the cuticle layer, exposing the cortex and making the hair shaft more prone to damage. Furthermore, the significant force and manipulation involved in backcombing can ultimately lead to TA. Finally, when combined with hairsprays or heat styling tools, backcombing can cause hair weathering if not done with care.^7^, ^8^, ^43^, ^60^^,^^61^ There is a condition, known as bubble hair deformity, characterized by the formation of air cavities within the hair cortex, primarily affecting females, that may be related to backcombing as well as any hairstyle involving heat styling tools. It is typically associated with the prolonged exposure of damp hair to high temperatures generated by blow dryers or styling devices. The high temperature causes hydrolysis of keratin within the hair shaft, leading to local air expansion and the formation of bubbles. While most reported cases of bubble hair deformity involve Caucasian patients, it has been suggested that there may be ethnic differences in susceptibility to this condition: for example, Asian hair has been shown to exhibit significantly more heat-induced protein loss compared to Caucasian hair, potentially due to structural differences in the hair cortex.^62^

**Fig. 3:**
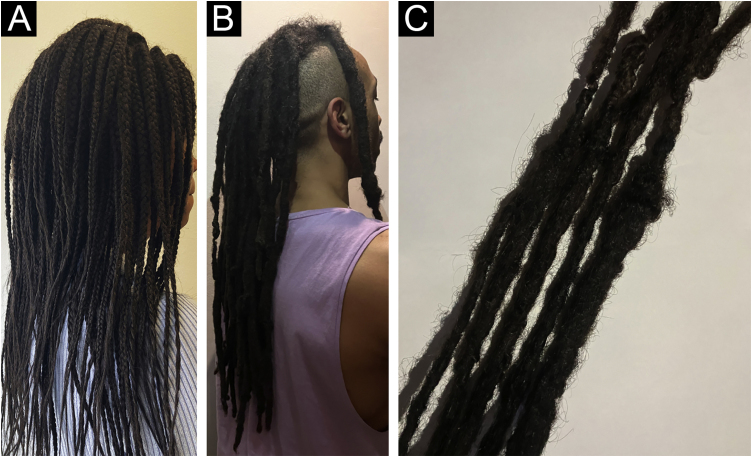
Different types of dreadlocks (A‒B). A close-up image (C) that shows how uncombed hair is twisted to form such clusters referred to as “locks”.

Finally, dreadlocks symbolize a prevalent method of hairstyling wherein uncombed hair is twisted to form clusters referred to as “locks”, which pose difficulties in detangling (Fig. 3A‒B). This trend is especially favored among younger individuals, particularly those with African hair, as it presents a natural, low-maintenance option for daily grooming without resorting to chemicals. The process typically entails dividing hair sections into two parts, twisting them, and using styling products like gel or beeswax to secure them. Without regular upkeep, dreadlocks may become permanently tangled and exert prolonged pressure on the scalp over the years. Concerning the impact of dreadlocks on hair health, they can contribute to TA due to the consistent scalp pressure, especially when extensions are incorporated, leading to additional strain on HFs and resulting in hair weathering over time. Furthermore, dreadlocks present challenges in moisture maintenance compared to straight hair because the tightness of the locks can impede the natural oil distribution along the hair shaft, resulting in dryness and potential fragility.^55^, ^57^, ^63^

## Conclusions

In summary, hair weathering is influenced by a wide range of exposomal factors such as medications, stress, UV radiation, water exposure, trauma, and hairstyling practices. These factors can lead to various effects on hair, contributing to its aging process. It's crucial to recognize these influences and take proactive measures to protect and fortify hair. The take-home-message is that by comprehending and addressing these factors, the authors can uphold optimal hair health and delay its aging. Looking ahead, it's imperative to conduct further research and foster collaboration among healthcare providers, researchers, and the beauty industry to deepen our understanding and mitigate the exposomal impacts on hair aging. This collective knowledge can drive the development of more advanced treatments and preventive strategies for a broader spectrum of hair aging-related issues.

## Authors’ contributions

Stephano Cedirian: Performed the research, analyzed the data, wrote the paper, approved the final manuscript as submitted and agreed to be accountable for all aspects of the work.

Ludmila Prudkin: Performed the research, designed the research study, analyzed the data, wrote the paper, approved the final manuscript as submitted and agreed to be accountable for all aspects of the work.

Bianca Maria Piraccini: Performed the research, analyzed the data, wrote the paper, approved the final manuscript as submitted and agreed to be accountable for all aspects of the work.

Julia Santamaria: Performed the research, designed the research study, analyzed the data, wrote the paper, approved the final manuscript as submitted and agreed to be accountable for all aspects of the work.

Jaime Piquero-Casals: Performed the research, designed the research study, analyzed the data, wrote the paper, approved the final manuscript as submitted and agreed to be accountable for all aspects of the work.

David Saceda-Corralo: Performed the research, analyzed the data, wrote the paper, approved the final manuscript as submitted and agreed to be accountable for all aspects of the work.

## Financial support

This study was not funded but the authors have been employed by ISDIN® to elaborate this project.

## Conflicts of interest

The authors have been employed by ISDIN® to write this paper.
